# Draft Genome Sequence of a Novel *Marinobacter* sp. Strain from Honolulu Harbor, Hawai‘i

**DOI:** 10.1128/genomeA.01354-16

**Published:** 2016-12-08

**Authors:** Xuehua Wan, Shaobin Hou, Siobhan L. Burns, Jennifer A. Saito, Stuart P. Donachie

**Affiliations:** a Advanced Studies in Genomics, Proteomics and Bioinformatics, University of Hawai‘i at Mānoa, Honolulu, Hawai‘i, USA; b Department of Microbiology, University of Hawai‘i at Mānoa, Honolulu, Hawai‘i, USA

## Abstract

*Marinobacter* sp. strain X15-166B^T^ was cultivated from sediment in Honolulu Harbor, Hawai‘i. The X15-166B^T^ draft genome of 3,490,661 bp encodes 3,115 protein-coding open reading frames. We anticipate that the genome will provide insights into the strain’s lifestyle and the evolution of *Marinobacter*.

## GENOME ANNOUNCEMENT

During an investigation of the effects of a molasses spill on the taxonomic diversity of culturable bacteria in Honolulu Harbor sediment, strain X15-166B^T^ was cultivated from sediment spread on ZoBell’s 2216E Marine Agar (MA) and incubated at 30°C. A BLAST comparison of 1,528 nucleotides of the strain’s 16S rRNA gene showed the nearest validly published neighbors (97% sequence identity) are *Marinobacter maritimus* CK47^T^ from seawater off the Subantarctic Kerguelen Islands and *Marinobacter psychrophilus* 20041^T^ from sea ice in the Canadian Basin (1, 2). *M. maritimus* grows at 4 to 37°C, while *M. psychrophilus* grows at 4 to 22°C. The mean annual seawater temperature at Honolulu is ~26°C (NOAA). Preliminary data suggest that X15-166B^T^ grows on MA up to ~37°C.

The genus *Marinobacter* hosts 40 species cultivated from marine and other saline habitats and is considered ubiquitous in the ocean (2–5). *Marinobacter* species have also been described as “opportunitrophs” able to survive under a range of conditions (6, 7). Since strain X15-166B^T^ appears on the basis of 16S rRNA gene nucleotide identity to be a novel *Marinobacter* species, we chose to sequence the strain’s genome to determine if it also has features that would enable survival in diverse habitats, e.g., phosphonate metabolism, acquisition of iron, or utilization of different terminal electron acceptors, such as one would find at different depths in marine sediments (5, 8).

Genomic DNA was isolated using the Wizard genomic DNA purification kit (Promega, USA) with additional cetyltrimethylammonium bromide (CTAB). Roche 454 GS FLX+ pyrosequencing generated 54.5 Mb of shotgun sequences and 55.3 Mb 8-kb paired-end sequences. Newbler 2.8 assembled the reads into 4 scaffolds containing 3,490,661 bp (scaffold *N*_50_, 3,483,170 bp), comprising 27 contigs (contig *N*_50_, 269.4 kb). Of the assembled bases, 99.82% have a base accuracy quality value above the Phred quality score of Q40. The genome’s G+C content is 59.7%.

The genome was annotated in the NCBI Prokaryotic Genome Annotation Pipeline (PGAP), the Rapid Annotation using Subsystem Technology (RAST) server, and Prokka 1.11 (9–12). PGAP identified 3,115 protein-coding genes, 43 tRNA-coding regions, and 110 pseudogenes. RAST identified 3,343 protein-coding open reading frames, 42 tRNA-coding regions, and 456 subsystems. RAST predicted that 20 proteins are phage components. RAST and the Kyoto Encyclopedia of Genes and Genomes (KEGG) identified the flagellar assembly system and chemotaxis system, and KEGG further predicted a two-component system involved in twitching motility, suggesting that *Marinobacter* sp. X15-166B^T^ can switch between flagellum-mediated swimming and pili-mediated twitching. Unlike in the genome of *Marinobacter aquaeolei* VT8 (a heterotypic synonym for *Marinobacter hydrocarbonoclasticus* SP17) (13, 14), in which 18 proteins were predicted to be involved in the siderophore subsystem, no such biosynthesis subsystem was identified in the genome of X15-166B^T^. However, siderophore-dependent iron acquisition ABC transporters, including FhuB, FhuC, and FhuX, and the siderophore-independent ferric uptake system, FbpABC (15), were predicted in X15-166B^T^ by BLASTP (*E* < 1e^−10^), suggesting that X15-166B^T^ may have lost the siderophore biosynthesis pathway and be a “cheater” that takes up others’ siderophores (16). The genome of this putative new species will provide insights into the lifestyle of X15-166B^T^ and the evolution of *Marinobacter*.

### Accession number(s).

This whole-genome shotgun project has been deposited at DDBJ/EMBL/GenBank under accession number MEIY00000000. The version described in this paper is the first version, MEIY01000000. The 16S rRNA gene nucleotide sequence has been deposited at GenBank under accession number KX808654.
